# Allostatic and Circadian Drives in Patients with Bipolar Disorder in Depressive Episodes or with Post-Traumatic Stress Disorder Versus Healthy Controls: Neuroendocrine Comparison Through Cortisol and 6-Sulfatoxymelatonin Overnight Urine Excretion

**DOI:** 10.3390/ijms27187993

**Published:** 2026-09-08

**Authors:** Valerio Dell’Oste, Matteo Gambini, Virginia Pedrinelli, Berenice Rimoldi, Lionella Palego, Gino Giannaccini, Laura Betti, Claudia Carmassi

**Affiliations:** 1 Department of Clinical and Experimental Medicine, University of Pisa, 56127 Pisa, Italy; gambinimatteo1996@gmail.com (M.G.); virginiapedrinelli@gmail.com (V.P.); bererimo@gmail.com (B.R.); lionella.palego@unipi.it (L.P.); claudia.carmassi@unipi.it (C.C.); 2 Department of Mental Health and Addiction, Azienda USL Toscana Nord-Ovest, 55100 Lucca, Italy; 3 Department of Mental Health and Addiction, Azienda USL Toscana Nord-Ovest, 57100 Livorno, Italy; 4 Department of Pharmacy, University of Pisa, 56126 Pisa, Italy; gino.giannaccini@unipi.it (G.G.); laura.betti@unipi.it (L.B.)

**Keywords:** bipolar disorder, depression, Post-Traumatic Stress Disorder, neuroendocrine axes, 6-sulfatoxymelatonin, Cortisol, Cortisol-to-aMT6s ratio, overnight urine

## Abstract

Neuroendocrine and circadian dysfunctions are thought to underlie bipolar disorder (BD) and could be implemented as markers of complex symptom conditions, including the presence of Post-Traumatic Stress Disorder (PTSD) comorbidity. The primary objective of this study was to investigate neuroendocrine profiles in different BD phenotypes through the measure of nocturnal urinary levels of Cortisol (stress-response factor) and 6-sulfatoxymelatonin (aMT6s; light/dark signal) in euthymic BD patients diagnosed with PTSD (PTSD group) or with major depressive episodes without trauma symptoms (DEP group). Both groups were compared against healthy controls (CTL). Nighttime urinary levels of aMT6s and Cortisol were analyzed by competitive ELISA, with ensuing values normalized for specific gravity. The Cortisol-to-aMT6s ratio (Cort/aMT6s) was calculated as an exploratory proxy for the homeostatic balance between HPA-mediated catabolic/coping activities and Melatonin-related reparative functions. Clinical severity and psychosocial activities were assessed by using mood HAM-D, YMRS, trauma IES-R and functioning WSAS scales. Results revealed distinct biological patterns: PTSD subjects showed increased nocturnal Cortisol compared to DEP and CTL groups, whereas DEP patients exhibited lower aMT6s levels and an elevated Cort/aMT6s ratio. Cortisol and aMT6s correlated positively in PTSD, while the ratio correlated positively with Cortisol solely in controls. Clinically, Cortisol correlated positively with IES-R and functional impairment (WSAS), whereas aMT6s correlated negatively with HAM-D and YMRS scores. Present findings suggest a possible neuroendocrine divergence in BD patients with depression versus PTSD: nocturnal Melatonin deficiency may be associated with depression, while HPA-axis hyperactivity and altered crosstalk between the two systems may characterize PTSD comorbidity, with the Cort/aMT6s ratio as a potential index of impaired daily functioning. These results could be promising for the use of biomarkers linked to allostatic and circadian responses within the BD spectrum.

## 1. Introduction

### 1.1. Clinical Background

Bipolar disorder (BD) is one of the most debilitating and life-threatening psychiatric conditions, primarily due to the chronic, unpredictable and alternating nature of its severe neurovegetative and behavioral symptoms. While the observation of mania and depression in the same individual has ancient Greek origins, scientific and medical interest in this mental illness has evolved very slowly over time. It was not until the 19th century, with Falret’s concept of “folie circulaire” [1] and the concurrent “folie à double forme” described by Baillarger (1854) [2], that concerns on BD as a medical condition were markedly revived. Furthermore, more than a century has passed since these concepts were introduced, evolving through Kraepelin’s separation of opposite mood states and schizophrenia [3] to the modern classifications of BD proposed in the DSM-5 (2013) [4], DSM-5R (2022) [5], and other diagnostic guidelines [6]. This also means that treatments for BD have taken a long time to become available, while still needing improvement and greater specificity with respect to the management of various clinical presentations and common overlaps with many other mental and somatic conditions [7,8]. Indeed, BD is now increasingly recognized as a psychiatric condition that extends beyond the classic alternation of manic and depressive episodes. It is characterized by profound mood instability, where euthymia frequently intertwines with a broad range of clinical manifestations, including depressive, hypomanic and manic phases, as well as comorbid anxiety and trauma-related syndromes, such as Post-Traumatic Stress Disorder (PTSD) [9,10]. This inherent heterogeneity became more evident with the introduction of a dimensional approach in clinical psychiatry, known as the mood disorder spectrum [11,12,13]. The mutable onset of symptoms contributes to the high frequency of initial misdiagnosis, a critical issue that often delays the implementation of optimal clinical care [14]. In addition to the higher mortality rate due to external factors [15], there is a common overlap of symptoms in BD across different psychiatric dimensions, alongside a high prevalence of co-occurring somatic syndromes [15,16]. It is therefore not surprising that there is a current growing interest in possible hypotheses related to its etiopathogenesis.

### 1.2. Etiopathogenesis and Molecular Biomarkers of Bipolar Disorder

Establishing a solid and comprehensive understanding of BD remains a fundamental objective for developing effective therapeutic approaches, reducing episode recurrency, and delaying disease progression. The present assumption is that this illness is founded on multifactorial causes, neurobiological, psychological and psycho-social, intertwined with one another [17,18,19]. The familiarity and biological grounds of BD have been explored, and neurobiological surveys have underscored the involvement of genetics, epigenetics, transcriptomics, proteomics and metabolomics at different levels over these last decades, affording support to a multidimensional vision of this mental disease. Many endeavors have been made to correlate these biochemical signatures with symptom clusters in the context of different polarities and features of episodes. The circularity that defines BD, the recurrence and alternance of mood episodes together with common sleep disturbances and difficulties in respecting daily activity during suitable time scheduling, has prompted researchers and clinicians to investigate circadian drives and their molecular effectors in patients [20]. Circadian drives represent the main hormonal patterns that act according to daily variations; essentially, periods of light and darkness due to the Earth’s rotation on its axis, which have evolved to adapt all living organisms to these environmental changes in a flexible way. The circadian system regulates the endogenous arousal/sleep cycle that generates the molecular bases of the “biological day” and the “biological night” through the action of fine-tuned neuroendocrine mechanisms [21]. These clockwork “regulators”, by establishing the diurnal alternation of specific molecular pathways, behave at the supramolecular level of other hormones such as insulin, glucagon, growth factors, and other signals, all of which follow the oscillations of endogenous biological clocks. Proteins encoded by biological clock genes are produced in the Suprachiasmatic Nucleus (SCN) of the hypothalamus (the “Master” clock) and in the periphery, such as in immune cells, promoting the release, over a period of approximately 24 h, of specific signals capable of synchronizing and calibrating their activity with “Zeitgebers” such as light and daily routines [22]. Circadian signals are even thought to represent the main molecular patterns involved in BD [23]. Most BD patients present disturbed sleep–wake cycles, eventually persistent also during the euthymic phases [24]. Sleep disturbances may include not only insomnia but also hypersomnia during the day, loss of alertness, difficulty in falling asleep during depressive episodes, poor sleep habits during mania, reduced sleep efficiency, and increased anxiety about falling asleep, resulting in disturbed or out-of-sync sleep–wake cycles [25,26,27]. Alloy et al. (2017) [25] have discussed the role of variants of the Master Clock genes in BD. It has been thus evidenced that dysfunctional circadian rhythms can be a trait marker of BD, being associated with mood episodes in BD [25,28]. Significant associations have been identified between BD and various genetic alterations within the molecular machinery of the Master Clock [29,30]. Specifically, haplotypes of core clock genes such as *Bmal1*, *TIM*, *PER3*, and polymorphisms of *CLOCK* have been linked to BD [29,31,32], as have those of regulatory kinases including *NR1D1* [33,34] and *ROR* genes [35,36]. Furthermore, enzymes acting at the crossroad between metabolism and circadian rhythms, such as GSK-3β—a key regulator of glycogen synthase and brain glucose homeostasis [37,38]—and CSNK1ε [39], have been significantly associated with the disorder. Notably, *TIMELESS* variants have been connected to specific symptom clusters involving suicidal behaviors [40]. Beyond genetics, epigenetic and transcriptomic studies have reported extensive alterations in BD [30,41]. Histone acetylation/deacetylation mechanisms, particularly the action of p300 histone acetyltransferase (EP300) on *CRY* and *PER* promoters [41], regulate Master Clock expression. Additionally, the NAD^+^-dependent deacetylase SIRT1 modulates the CLK-BMAL1 complex [42]. SIRT1 activity depends on NAD^+^ concentrations, which are linked to the kynurenine (KYN) shunt—a tryptophan (TRP) metabolic pathway opposite to serotonin (5-HT) and Melatonin formation [43,44]—involved in mood disorders [45,46,47,48]. At the RNA level, changes involving miRNA and alternative splicing [49] likely correlate with proteomic and metabolomic discrepancies in BD [50,51]. Metabolomic research has focused on Melatonin synthesis from the TRP/5-HT axis in counterbalance with the KYN shunt [52,53,54,55]. Significant impairments in Melatonin and its metabolite 6-sulfatoxymelatonin (aMT6s) have been observed in the saliva, plasma, and urine of patients with depression and BD [53,55,56,57,58,59,60,61,62,63,64,65,66,67,68,69], although some findings remain to be fully confirmed. Melatonin synthesis occurs in the pineal gland [70,71] via a multi-step process regulated by the SCN. This circuit integrates the activity of Master Clock gene products [72] with the light/dark “Zeitgeber”, involving a retinal pathway through the hypothalamic paraventricular nucleus (PVN) and Superior Cervical Ganglion (SCG) to trigger the enzymatic conversion of N-acetyl-5-HT (NAS) into Melatonin via Hydroxy indole-*O*-methyltransferase HIOMT [73]. This fine-tuned regulation is interrelated with peripheral clocks, particularly in immune cells, allowing for peripheral investigations as a window on brain function [74,75,76]. Beyond its chronobiological role, concurring at harmonizing metabolism with the dark/light “Zeitgeber”, Melatonin is also endowed with intrinsic antioxidant properties, directly acting as a free radical scavenger compound, representing a resilience molecule against oxidative stress [77]. Nocturnal Melatonin production intersects with the hypothalamus–pituitary–adrenal (HPA) axis, which operates during the daylight period [78,79,80,81]. The morning Cortisol surge follows an opposite release pattern to the Melatonin peak [82,83,84]. The HPA axis also governs the “fight-or-flight” stress-adaptive response, a brain–body process aimed at coping with environmental stressors [85,86] at the cost of allostatic “wear-and-tear” [87,88]. Disruptions in the HPA axis and Cortisol levels have been extensively documented in BD during both euthymic and active clinical phases [89,90,91,92].

### 1.3. Rationale and Objectives of the Study

Due to these features, the Melatonin and HPA systems can be evaluated simultaneously to gain insight into the balance of the circadian and allostatic drives in BD patients, particularly in stratification surveys, also considering comorbidity with other severe psychiatric conditions as Post-Traumatic Stress Disorder (PTSD) [93,94,95]. This has also been related to an increased risk of developing PTSD, which, indeed, is often associated with BD and is also characterized by hyperarousal symptoms, HPA axis dysfunction [96], nocturnal troubles, REM sleep disturbances, and a perturbed Melatonin system [97,98]. While patients with BD often exhibit circadian and HPA axis abnormalities, a comprehensive understanding of their prognostic impact requires a precise classification based on specific clinical symptoms and molecular markers. The accurate identification of these dysfunctions may help pinpoint specific vulnerabilities in BD, potentially preventing progression toward treatment-resistant states and the chronic relapse patterns typical of the disorder.

Ultimately, this approach aims to optimize therapeutic interventions and enhance patient quality of life. In this framework, the present study aimed to search for distinctive urinary excretion of Cortisol and the main final Melatonin metabolite 6-sulfatoxymelatonin sulfate (aMT6s) [99]. For this purpose, overnight urine samples collected during a period of about 8 h were evaluated in a cohort of BD patients with a PTSD diagnosis during euthymia or with current major depressive episode diagnosis without PTSD. The Cortisol-to-aMTs6 (Cort/aMT6s) ratio was calculated as a combined index of the two systems. These data were then compared across groups and against healthy controls to distinguish biochemical profiles and determine significant correlations with clinical symptoms.

## 2. Results

After the recruitment procedure, our subjects’ sample finally encompassed 59 individuals, a total of 20 BD patients in euthymic phase with full-blown PTSD symptoms (PTSD group), 20 BD patients with a moderate-to-severe major depressive episode (MDE) without PTSD signs (DEP group) and 19 healthy controls (CTL group). Sociodemographic features and clinical psychometric results were described in our previous investigations [48,100]. The average age of the participants was 45.02 ± 16.13 years, prevalently including women, and there were no differences in age or sex among the three groups. All patients were recruited under a stable psychopharmacological treatment in terms of administered psychotropic drugs and dosages for at least one month before recruitment in the survey, as established by the inclusion criteria. This aspect is described in more detail in other previous works [48,100]. Psychotropic medication consisted mainly of antidepressants, mood stabilizers, anti-psychotics and benzodiazepine drugs, administered alone or in associations, without an appreciable difference among patients’ groups [48]. The mean duration of BD accounted for 15.18 ± 12.75 years, while the mean value of years after the traumatic event in patients with PTSD was 7.37 ± 12.52 years, with 60% reporting a highly stressful event occurring in the last year.

Table 1 reports results concerning the comparisons between the different urinary parameters and analytes in the three groups of subjects under investigation. All recruited subjects had no relevant amounts of glucose, proteins, bilirubin, urobilinogen, blood, ketones, and nitrites in urine samples, with 0 cells/μL to 25 cells/μL leukocytes, the latter values present in ~8% of them, who, however, did not present any relevant clinical symptoms at the time of the investigation. No significant between-group difference was observed in total volume as well as in the pH values and specific gravity (SG) of urine collected during the night and early morning after getting up. The functioning of the neuroendocrine HPA axis appraised by urinary Cortisol, excreted as total μg in the same nighttime samples used for the analysis of aMT6s, showed significantly higher levels of the glucocorticoid in PTSD vs. DEP patients and CTL subjects.

We compared herein aMT6s in overnight urine as total excreted amounts in μg during the ~8 h monitoring period, obtaining markedly lower concentrations of this parameter in the DEP group vs. the CTL one, together with significantly decreased levels, albeit to a lesser extent, vs. euthymic BD patients with PTSD.

Notably, when the ratio between total urinary μg of Cortisol and aMT6s was compared, only DEP patients reported a statistically significantly increased ratio compared to CTL subjects. The ratio was variably higher in the PTSD group without reaching statistical significance in respect to both the CTL and DEP ones.

**Figure 1:**
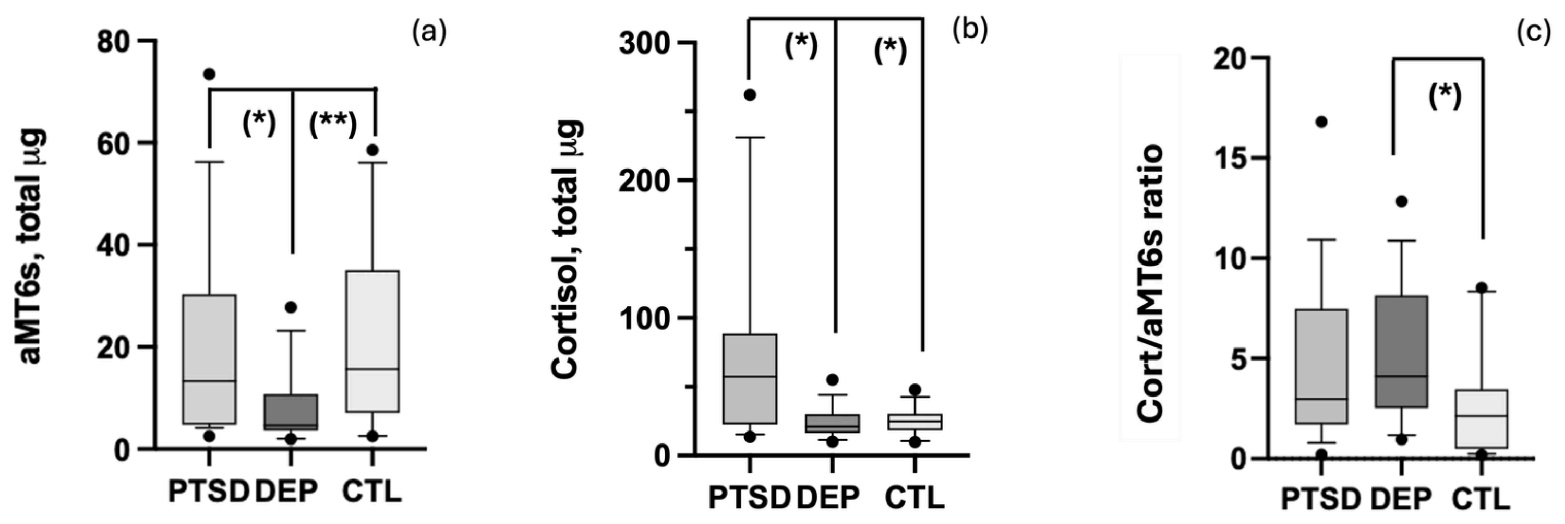
Comparisons between aMT6s, Cortisol and Cortisol/aMT6s ratios in urine samples from the three groups under investigation. Data are presented as box and whiskers, medians and 10–90th quartiles of aMT6s (**a**), Cortisol (**b**) and Cortisol/aMT6s ratios (**c**), measured in nighttime/getting-up urine of BD patients with PTSD (n = 20, PTSD group), with major depressive episodes (MDE) (n = 20, DEP group) and healthy controls (n = 19, CTL group); (**): significant Kruskal–Wallis analysis, *p* < 0.01; (*): significant Kruskal–Wallis analysis, *p* < 0.05.

**Figure 2:**
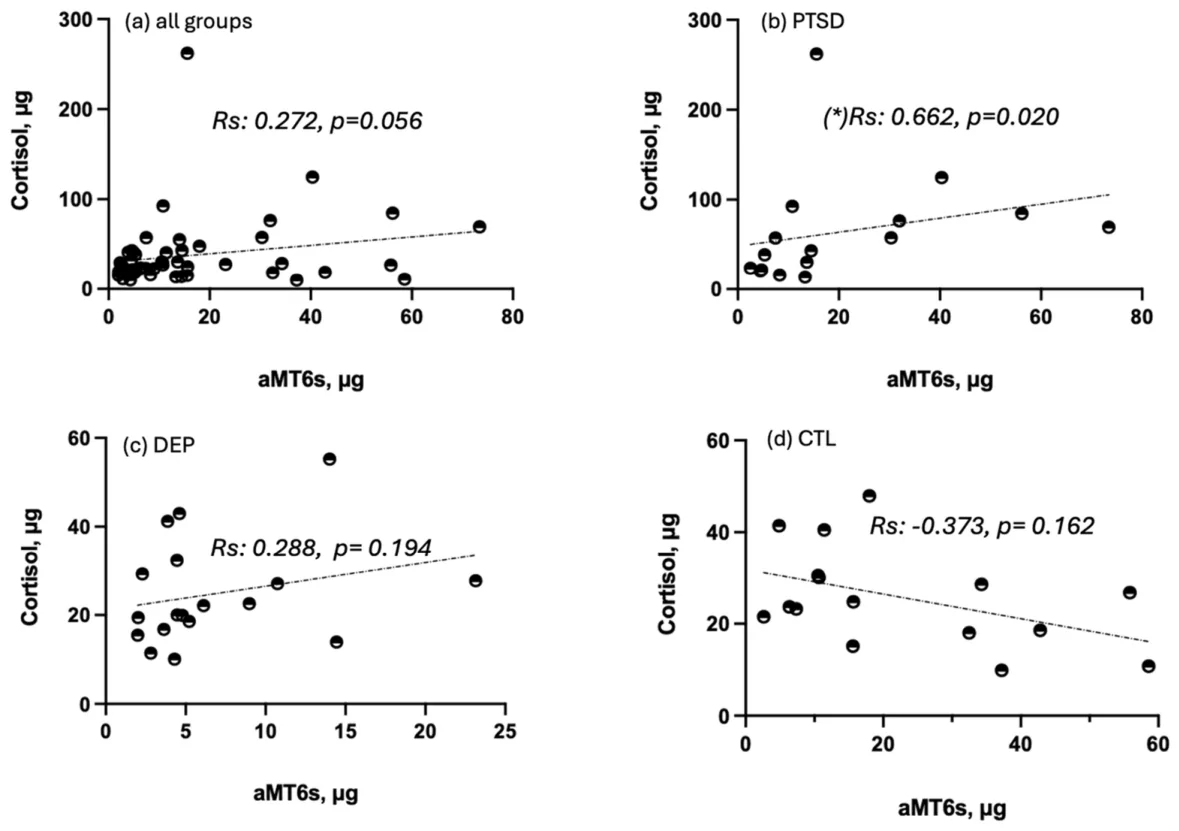
Spearman correlations between nighttime urinary Cortisol and aMT6s, including the entire study population (**a**) and each clinical group separately, PTSD (**b**), DEP (**c**), and CTL (**d**). Rs = Spearman correlation coefficient, *p* = adjusted values by FDR test. (**a**,**c**,**d**): not significant, *p* > 0.05; (**b**): (*) significant association passing the FDR test, *p* < 0.05. The dotted linear regression lines are displayed for visualization of the overall directional trend and were not used for statistical inference.

Figure 1 reports comparisons of aMT6s, Cortisol and their ratios, in the three groups of subjects recruited for the study, to visualize results shown in Table 1. Figure 2 reports the Spearman correlations obtained between the two biomarkers of the two circadian and allostasis systems, aMT6s and Cortisol, in urines from all subjects (Figure 2a) or in the three-group subjects, separately (Figure 2b–d): Figure 2a depicts the positive trajectory of the Spearman correlation in all subjects, not significant after FDR correction (trend, *p* = 0.056); Figure 2b–d shows a significant positive correlation obtained exclusively in the PTSD group.

**Figure 3:**
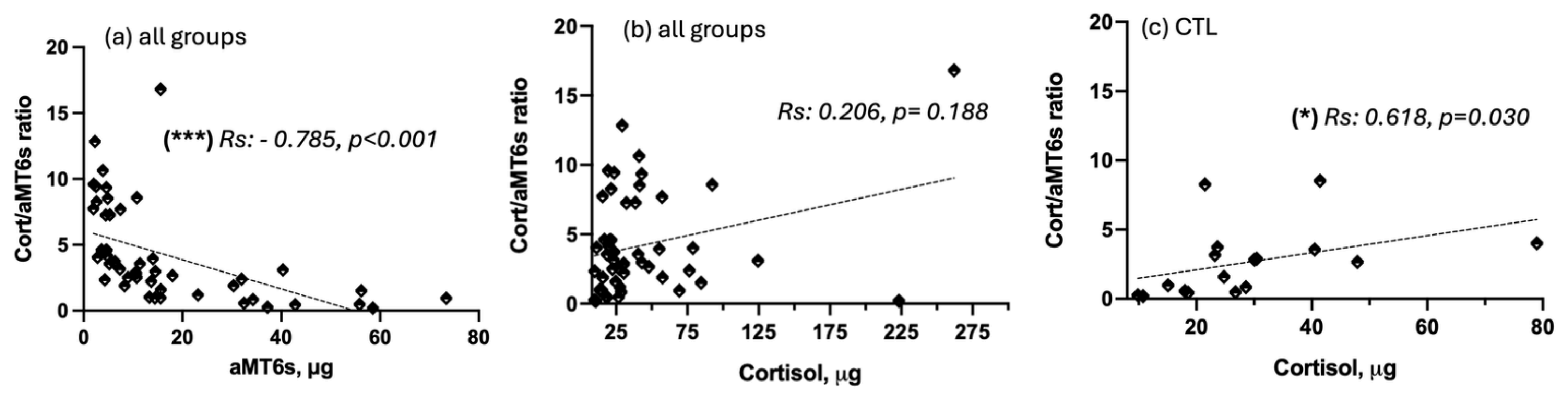
Spearman correlations of nighttime urine Cort/aMT6s ratios vs. aMT6s (**a**) or vs. Cortisol (**b**), including the entire study population; in (**c**) the correlation between the Ratio and Cortisol in CTL subjects is depicted. Rs = Spearman correlation coefficient, *p* = adjusted values by FDR test. (**a**): (***): strongly significant correlation, *p* < 0.001; (**b**): not significant, *p* = 0.188; (**c**): (*): significant correlation, *p* = 0.030. The dotted linear regression lines are displayed for visualization of the overall directional trend and were not used for statistical inference.

Figure 3 depicts instead the relationships between the Cort/aMT6s ratios vs. each examined biological parameter, Cortisol and aMT6s. In detail, Figure 3a shows a statistically significant negative correlation among the ratios and aMT6s obtained when analyzing all subjects; this negative correlation was found significant also in each of the three groups separately, PTSD: Rs: −0.573, *p* = 0.007; DEP: Rs: −0.758; *p* < 0.001; and CTL: Rs: −0.950, *p* < 0.001.

Conversely, the positive correlation between Cort/aMT6s ratios and Cortisol in all groups did not reach the statistical significance (Figure 3b). A statistically significant positive correlation between the Cort/aMT6s ratio and Cortisol was reported when the CTL group was analyzed separately (Figure 3c). Instead, no statistically significant correlation between the Cort/aMT6s ratio and Cortisol was obtained in the DEP (Rs: 0.33, ns, *p* = 0.190) or PTSD (Rs:0.037, ns, *p* = 0.700) groups separately.

Some main significant correlations between biological parameters and the clinical psychometric results were also reported (Table 2). Overnight urinary Cortisol was found to be significantly and positively correlated with IES-R total and domain scores; conversely, overnight urinary aMT6s was significantly and negatively correlated with both the HAM-D and YMRS total scores for current depression and mania symptoms, as well as with the “Work functioning impairment” item of the WSAS. The Cort/aMT6s ratio was significantly and positively correlated with HAM-D and YMRS total scores, as well as with the IES-R “Intrusion” and “Avoidance” domain scores. Moreover, the ratio also markedly and positively correlated with the “Work functioning impairment” as well as with the “Home management impairment” items of the WSAS.

## 3. Discussion

As far as we know, this study provides the first evidence regarding overnight and early-morning urinary profiles of Cortisol (main HPA signal) and aMT6s (light/dark hormone) as potential biomarkers of BD clinical heterogeneity. Indeed, we compared these parameters across specific BD clinical states—namely, during a major depressive episode (DEP group) or in the presence of PTSD (PTSD group)—against a healthy control cohort (CTL group). Also, the originality of the present investigation fits in with the effort to stratify BD patients, not only at the clinical but also at the neuroendocrine and biochemical standpoints, an approach recognized as one of the most relevant in the healthcare of psychiatric patients. While etiopathogenesis of BD remains almost unknown by now, much progress has been made by highlighting, as previously reported, a key role of altered circadian and HPA responses [101]. Moreover, previous clinical studies have pointed out that impaired circadian activities in BD patients can reflect different mood states and illness stages [27,102,103]. Despite this framework, the biological correlates of trauma-related symptomatology in BD have been relatively less explored and our previous and current studies aim at filling this gap [48,100,104]. Patients with BD are more likely to develop PTSD symptoms following exposure to extremely stressful situations, such as actual death or the threat of death, serious injury, or sexual violence, whether experienced personally or witnessed to have happened to a loved one [93,95,105,106]. As aforementioned, the two neuroendocrine pathways explored herein have been prevalently appraised separately in biological investigations on BD, although an evaluation of one in respect to the other is expected to provide additional insight in patient phenotyping [107]. Most of these investigations have measured plasma or saliva levels of Melatonin or HPA players at different times of the day in patients with mood disorders [108,109,110,111,112,113,114,115,116]. Only a few authors have investigated these neuroendocrine mediators within the same patient cohort, reporting altered—albeit not yet conclusive—release profiles, thus supporting the need for further research along this path [117,118].

For this purpose, we have chosen to directly measure these signaling molecules in non-invasive samples as urine, a stable biological fluid. Melatonin, derived by 5-HT metabolism via the TRP methoxy-indole pathway, is mostly catabolized by cytochrome P450 in the liver, transformed into 6-hydroxymelatonin (6-OH-MLT) and subsequently to aMT6s by phase-II sulfotransferases, principally by the SULT1A1 and SULT1C4 isoforms [119], thus generating the final product of this path excreted in the urine [120]. The concentration of urinary aMT6s correlates well with the total level of Melatonin released into the bloodstream [121,122], and overnight/early-morning aMT6s reflect the sum of nocturnal pineal Melatonin secretion [123,124]. Thus, aMT6s can be easily evaluated in urine samples from patients, and its levels are considered representative of the total Melatonin release from the pineal gland during the monitoring daily period [59,62,125]. As regards urinary Cortisol assessment, this is considered a well-established routine laboratory examination of HPA axis activity. Collecting a 24 h urine sample is much less invasive than repeated blood draws or collecting separate urine samples multiple times throughout the day. Even if in 24 h urine sampling the measured concentration is the sum of the daily concentration of hormones, without giving their profiles and shifts during the day, this procedure is considered a gold-standard method in neuroendocrinology [126]. However, it has been reported that 24 h urine specimens can be a source of analytical inaccuracy if guidelines are not strictly followed [127], which can represent a critical aspect during sample gathering of psychiatric outpatients, as in the present case. Furthermore, during the night, Melatonin levels rise significantly while Cortisol amounts are very low; Cortisol begins to increase around 4:00 a.m. and reaches its maximal peak a few hours later, between 7:00 and 9:00 a.m., when Melatonin levels are declining. Thus, urine samples collected from the past evening to the next morning contain the sum of the overnight release of aMT6s and its acrophase, slightly delayed relative to that of its parent compound, together with that of low nighttime Cortisol and its first-morning arousal, permitting us to catch the night-to-day phase shift toward the Cortisol Awakening Response (CAR) at its maximum within 30–45 min after getting up [128]. Overnight and early morning urine samples in BD patients are therefore supposed to reveal Cortisol additional or intrusive excretion during this daily period. For these reasons, both biochemical parameters were analyzed in overnight urine samples also collected upon waking in the morning, easier to handle accurately than 24 h specimens and more informative for our targets. Moreover, urinary aMT6s and Cortisol values were here normalized in respect to specific gravity, a valuable and inexpensive procedure which correlates well with the more common correction for creatinine [129,130,131]. Concurrently, urine sample quality and suitability were monitored using an automated 10-parameter urinalysis system, which measures specific gravity alongside all major urinary parameters. This procedure prevented the inclusion of inappropriate samples and minimized analytical interferences. Furthermore, it allowed us to reliably rule out acute somatic, metabolic, inflammatory, or microbial conditions in the recruited subjects at the time of collection.

The results obtained through this study’s design deserve interest, as they reveal a neuroendocrine dissociation between the Melatonin system and HPA axis function, showing opposite features across different symptomatic phases of BD: subjects currently depressed without PTSD showed lower levels of nocturnal–early morning urinary aMT6s compared to those who were euthymic with PTSD, and to a greater extent compared to healthy controls. Concomitantly, higher levels of nocturnal–early morning urinary Cortisol were found exclusively in euthymic BD patients with PTSD compared to BD patients with MDE and healthy volunteers. The Melatonin findings support the divergence between an MDE condition and PTSD with respect to TRP metabolism previously observed in BD patients [48]. In particular, low 5-HT was not followed by higher quinolinic acid (QUIN) in the plasma of PTSD patients as in the depressed ones, indicating a diverse regulation of TRP fates in these two conditions [48]. Notably, in the present investigation, nocturnal urinary aMT6s levels in PTSD patients were found to be comparable to healthy controls. This suggests that reduced circulating 5-HT in euthymic BD patients with PTSD may rather result from altered networks or from intracellular–extracellular fluxes rather than decreased production, more characteristic of depressed patients [48,122,132]. It is well known that Cortisol, directly or indirectly, induces TRP metabolism towards the KYN shunt [85,133]; however, in our patients with PTSD, in whom elevated levels of the HPA hormone were found, we merely observed an increase in the QUIN/KYN ratio, presumably due to different inductions of the various metabolic branches derived from KYN and different recruitment of KYN itself [48]. Thus, present data provide further support to the “classical” reduction in the indole-conserving pathway of TRP metabolism vs. the KYN shunt in the DEP group, while in the PTSD group, the reduced morning plasma 5-HT [48] combined with higher urinary overnight Cortisol implies a peculiar metabolic profile belonging to this subgroup of BD patients.

In the context of a hyperarousal state, as seen in PTSD patients who show perturbed vigilance centers in the brainstem and mesencephalic nuclei, a deregulated HPA axis with diminished immunosuppressive and immunomodulatory functions may contribute to chronic low-grade inflammation. This condition is defined by impaired glucocorticoid (GC) receptor sensitivity and blunted GC signaling, a molecular configuration linked to allostatic overload and chronic disease onset [134,135,136]. Other authors, rather, reported that impaired HPA function in the long term can enhance glucocorticoid negative feedback [136,137], frequently leading to lower circulating Cortisol levels in traumatized subjects when compared to controls [136,138,139]. These two HPA patterns have both been found possible in PTSD cohorts [96,140]. As regards our sample, we have predominantly investigated subjects with a traumatic event that occurred within the last year from the beginning of the study, which could explain the observed relative hyperreactivity of the HPA axis. Persistent high Cortisol levels can be a precursor to a blunted HPA axis in line with a stress-response model of “wear-and-tear” [141]. Abnormal HPA function can underlie the risk of developing PTSD: prospective studies indicate that individuals vulnerable to developing PTSD are characterized by dysregulations at different levels of the glucocorticoid signaling cascade [142], including lower circulating Cortisol levels prior to [143] and in the early aftermath [142,144] of trauma exposure, implying altered time-dependent dynamic shifts in the glucocorticoid response in these subjects. Different types of traumatic event experienced and/or co-morbidities would also affect results, as previously observed [138,140]. In our investigation, overnight Cortisol levels presumably reflect a higher release of this hormone in relation to the after-trauma re-experienced symptoms [145].

Our study also reported significantly higher Cort/aMT6s ratios in the DEP group only. The ratio between these two main SCN neuroendocrine players in urine samples has been previously evaluated in psychiatric illness, reporting lower aMT6s/Cort ratios (corresponding to higher Cort/aMT6s values) in depression and psychosis [146,147,148], in line with the present results. These authors, however, observed that this index was not specific enough, being dependent on many patients’ confounding factors. However, by performing additional analyses with the aim of better understanding the relationship between the two neuroendocrine systems in BD, we notably found that overnight urinary Cortisol was found to be positively correlated with aMT6s, exclusively within the PTSD group. Thus, these two hormones, which should operate in phase opposition, appear to act in synchrony in BD patients with trauma. This finding is quite surprising and interesting since it suggests that the nocturnal hormonal regulation in PTSD seems not only altered at the quantitative level, but also at the qualitative one. There could be, on one hand, a possible allostatic coupling with “reactive” and compensatory activity of the Melatonin system in the PTSD group, where restorative physiological systems would attempt to counteract the nighttime HPA elevated activity [149,150]. On the other hand, a potential loss of the SCN ability to distinguish between rest and arousal stages can also be hypothesized. It is possible that in patients with BD and comorbid PTSD, the urinary aMT6s peak is delayed toward the latter part of the night [20], resulting in a steeper release profile compared to healthy controls, who typically exhibit a broader nocturnal distribution. To better understand the respective contributions of the arousal/stress system and restorative sleep to the ratio across the different BD patients, we performed supplementary correlations between the Cort/aMT6s ratios and Cortisol or aMT6s. In this case, too, we reported divergent patterns: a significant positive correlation between the ratio and urinary Cortisol was found only in the CTL group; conversely, the ratio always showed negative correlations with aMT6s, in all subjects and groups separately.

**Figure 4:**
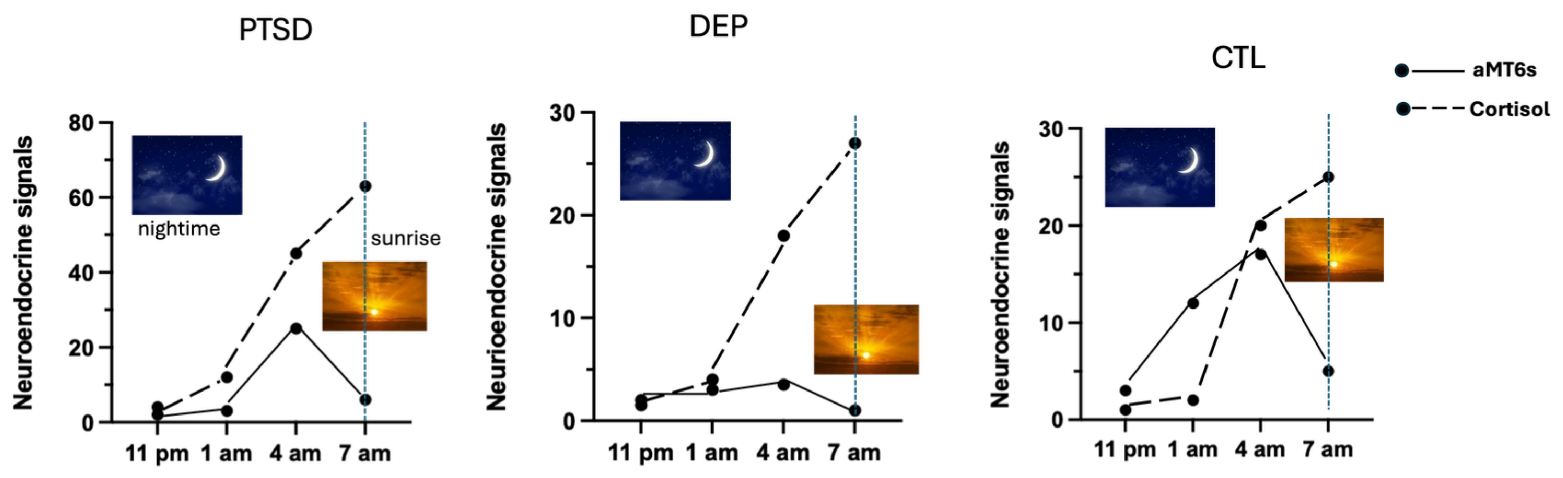
Hypothetical model of Cortisol and aMT6s excretion at different nocturnal hours in the three groups under investigation. Neuroendocrine signals (*y* axis): Putative and schematic Cortisol and aMT6s outputs (μg) excreted during the night at 11 p.m., 1 a.m., 4 a.m., and 7 a.m. (*x* axis). The higher y axis scale in the PTSD graph depicts the increased Cortisol levels reported in this specific condition vs. the DEP and CTL ones.

This suggests that nocturnal urinary Cortisol and Melatonin levels would vary differently with respect to each other in patients with BD, pointing to distinct underlying mechanisms across clinical subgroups. Specifically, the altered ratio in the DEP group appears primarily driven by disrupted Melatonin release, whereas in the PTSD group, more complex interactions between the two systems may account for the observed HPA axis hyperreactivity. Notably, in these series of correlations involving biological parameters, we observed Rs values of moderate-to-high impact, in accordance with Cohen’s criteria [151]. Figure 4 describes and resumes a hypothetical model showing some indicative theoretical profiles, based on the results and what has been discussed above.

Correlations of clinical outcomes provide further support for the neuroendocrine discrepancy observed among BD patients when considering the HPA axis function in stress coping and the circadian, antioxidant and restorative activity of Melatonin during sleep/night. Urinary Cortisol correlated positively with the IES-R total and domain scores, whereas aMT6s negatively correlated with HAM-D and YMRS total scores. The latter also negatively correlated with the WSAS item-1 score, suggesting that reduced release of Melatonin in the nighttime may indicate relevant disruption of occupational activity and significant consequent social impairment. Interestingly, the Cort/aMT6s ratio also positively correlated with both HAM-D and YMRS rating total scores, as well as with the IES-R Intrusion and Avoidance domain scores. The Intrusion domain is linked to the severity of intruding, unbidden re-experiencing of the traumatic events by memories, thoughts, flashbacks and nightmares; the Avoidance domain is instead more closely linked to evading cognitive constructs and behaviors related to events, activities, or anything else connected to the traumatic event(s), significantly impacting the PTSD patients’ quality of life. Thus, the altered Cortisol-to-Melatonin balance in BD during major depression would stem from nocturnal Melatonin suppression, proportional to mood severity. Conversely, the ratio’s positive correlation with trauma-response severity in the PTSD group would, rather, reflect a possible atypical coupling between the two neuroendocrine axes. The correlations among the ratio and WSAS item-1 and item-2 suggest that a dysregulated interplay between nocturnal Cortisol and Melatonin may be associated with daily work and home functioning. These findings suggest an immediate clinical implication, since the Cort/aMT6s ratio displays potential for identifying states of severe maladaptation and metabolic distress within the BD population. They also imply that this neuroendocrine parameter is not selective per se but can provide more information when considering the whole framework, reflecting the organism’s ability to shift from arousal and catabolism to sleep, rest and recovery.

The present investigation has limitations deserving mention, due to its cross-sectional design and small sample size, and the lack of an additional control group composed by euthymic BD patients without comorbidities. Methodological issues may also have influenced the results, especially regarding Melatonin dosage [152], although urinary aMT6s measurement is considered a robust and reliable test in the scientific community [122,153]. Further limitations are due to the enrollment of patients treated with psychotropic drugs, even if stable over time, compared to untreated controls, as already discussed [48,100]. Moreover, other parameters such as smoking behavior, physical activity, dietary habits, and other specific lifestyle variables were not assessed in this study. Furthermore, we could not assess sleep phase quality using specific psychometric tools within our sample cohort. Although sleep disturbances represent core symptoms of PTSD and depression assessed by the psychometric instruments used in the current research, future studies including more specific tools focused on sleep quality are warranted. While these limitations should be considered, the study offers a valuable inquiring framework aimed at generating novel hypotheses to guide future investigations.

## 4. Materials and Methods

### 4.1. Study Design and Participant Recruitment

This study employed a cross-sectional, case–control design. Participants were recruited from adult outpatients and inpatients at the Psychiatric Clinic of the University of Pisa (Department of Clinical and Experimental Medicine, Santa Chiara University Hospital—AOUP). The clinical cohort was firstly stratified into two distinct groups based on specific symptomatic clusters:

• PTSD Group: Bipolar patients in a euthymic phase with a current comorbid diagnosis of PTSD.
• DEP Group: Bipolar patients experiencing a major depressive episode (MDE) without PTSD.
• CTL Group: A control group of healthy volunteers with no personal or familial history of psychiatric disorders.

Inclusion criteria for patients were as follows: age between 18 and 65 years; history and diagnosis of bipolar disorder (BD) according to the DSM-5TR criteria, currently in a euthymic phase (only for the PTSD group) or in a MDE without concomitant PTSD diagnosis (only for the DEP group); for the PTSD group, a current diagnosis of PTSD according to the DSM-5TR criteria established at least one month after the traumatic exposure to exclude Acute Stress Disorder; enrollment at the first visit or follow-up control for outpatients or the first day of hospitalization for inpatients, before any clinical intervention or therapy variation applied at the time of clinical assistance and start of the study, thus comprising only subjects under a stable psychotropic treatment for at least one month; and acceptance of the protocol and signature of informed consent.

Exclusion criteria included: Age <18 or >65 years; history of significant neurological or systemic medical conditions, e.g., neurodegenerative diseases, multiple sclerosis, endocrine/metabolic dysfunction, inflammatory or autoimmune disorders, and cardiovascular disease; alcohol or substance use disorders within the previous six months; current pregnancy; and inability to provide informed consent. For the CTL group: the same exclusion criteria of patients besides the absence of any lifetime history or current diagnosis of psychiatric disorders according to DSM-5TR.

Upon enrollment, all subjects underwent a comprehensive clinical evaluation by experienced physicians and trained psychiatrists, utilizing semi-structured interviews and validated psychometric scales. Nocturnal urine samples were collected to quantify Cortisol and the Melatonin metabolite aMT6s. Urine samples were gathered at the ward of the Psychiatric Clinic of the University of Pisa for inpatients, assisted by the nursing staff, or delivered to the outpatient service of the Psychiatric Clinic by outpatients and controls. The study was conceived and conducted in accordance with the Declaration of Helsinki. All procedures were approved by the Ethics Committee of the University Hospital of Pisa (Protocol No. 19299/21).

### 4.2. Psychometric Assessment

Participants underwent a comprehensive psychopathological evaluation conducted by trained psychiatrists through clinical interviews and a battery of psychometric instruments, including both clinician-rated scales and self-report questionnaires. Socio-demographic and clinical data were systematically recorded in a dedicated Case Report Form (CRF). The diagnostic assessment was performed using the Structured Clinical Interview for DSM-5 (SCID-5) [154], a semi-structured tool designed to establish psychiatric diagnoses according to DSM-5 criteria. The SCID-5 consists of ten independent modules that guide the clinician through a systematic evaluation of symptomatic criteria. To quantify current symptom severity, the following instruments were employed:

• The Hamilton Depression Rating Scale (HAM-D) [155], a 21-item clinician-rated scale used to assess the severity of depressive symptoms. While the full version was administered, the first 17 “nuclear” items were used to determine the total score, with established cut-offs: severe (>25), moderate (18–24), mild (8–17), and no depression (<8).
• The Young Mania Rating Scale (YMRS) [156], an 11-item instrument evaluating manic symptomatology over the previous 48 h. Severity is assessed through both patient self-report and clinician observation. Notably, four items (irritability, speech, thought content, and disruptive behavior) are weighted double to account for potential poor cooperation in severely ill patients.
• The Impact of Event Scale-Revised (IES-R) [157]: A 22-item self-report scale measuring PTSD core features such as intrusion, avoidance, and hyperarousal relative to the previous week. A total score exceeding 32 serves as the diagnostic cut-off for PTSD.
• The Work and Social Adjustment Scale (WSAS) [158], to assess the global functioning impairment and disability. This five-item scale quantifies the impact of symptoms on work, home management, social and private leisure activities, and interpersonal relationships over the previous week. Each item is rated on a 0–8 scale, with a maximum total score of 40, demonstrating high internal consistency (α = 0.70–0.94).

### 4.3. Biochemical Assays

#### 4.3.1. Chemicals, Reagents and Instruments

All chemicals and reagents used for the investigation were of the best purity and quality, in agreement with the gold-standard guidelines of analytical laboratories. All solutions and buffers were prepared using an ultrapure HPLC gradient-grade milli-Q water (18.5 MΩ cm resistivity), obtained by means of a Simplicity Millipore Apparatus (Millipore S.A.S., Merk group, Molsheim, France) equipped with an UV-lamp and a 0.2-μm filter to exclude particles and biological agents. A PST-60HL plate thermo-shaker (Biosan, Riga, Lativia) was used for sample incubations in ELISA assays, when required. Absorbance at 450 nm for aMT6s assay was appraised using a 96-well plate spectrophotometer (MultiSkan FC ThermoScientific, Thermofisher Scientific, Waltham, MA, USA). For Cortisol detection at λ = 405–412 nm, the multimodal Enspire reader was employed (PerkinElmer, Waltham, MA, USA).

#### 4.3.2. Urine Sample Collection and Storage

Nocturnal urinary levels of the Melatonin metabolite aMT6s and Cortisol were determined from 8 h overnight and first-morning urine voids. Each outpatient and healthy control was provided with a standardized graduated collection vessel and a smaller aliquoting flask, along with detailed instructions for home collection. The protocol required participants to void their bladder between 10:30 and 11:00 p.m. (discarding this initial sample) before retiring for the night. All subsequent urine produced during the night, including any nocturnal voiding and the first morning when getting up, typically between 6:00 and 7:00 a.m., was pooled into the graduated container. For inpatients, the same standardized procedure was performed under the supervision of the nursing staff. Upon completion of the ~8 h collection period, the total volume was recorded, and a representative aliquot was transferred to the secondary flask, labeled with the participant’s unique identification code. After collection and subsequent delivery to the Psychiatric Unit, samples were then transported under controlled conditions to the Biochemistry laboratory of the Department of Pharmacy, University of Pisa, joined to the Psychiatric Clinic for laboratory analytical activities, where all laboratory procedures were carried out. Initial urinalysis was performed using an automated Arkray Aution-micro spectrophotometer and 10 parameter Aution sticks (Arkray Italia S.r.l., Vimercate, MB, Italy) to screen glucose, protein, bilirubin, urobilinogen, pH, specific gravity (SG), blood, ketone bodies, nitrites, and leukocytes. Subsequently, urine samples were centrifuged at 2000× *g* for 5 min at room temperature. The resulting supernatant was aliquoted into Safe-Lock Eppendorf tubes and stored at −80 °C until immunometric assay.

#### 4.3.3. ELISA Assay for Urinary 6-Sulfatoxymelatonin (aMT6s) 

Urinary aMT6s levels were quantified using a competitive ELISA kit (IBL International GmbH, Hamburg, Germany). This method was chosen for its high accuracy and presence of control samples and relevant specificity for the target analyte by cross-reactivity with other indoles <0.001%, as well as good precision provided by inter- and intra-assay variations of 6–17%. Furthermore, the analytical performance of the kit shows a high correlation with the LC-MS methodology. The day of assay, thawed urine samples, blanks (non-specific binding and other reagent controls), standards (1.7–420 ng/mL), and quality controls were diluted 1:50/100 (*v*:*v*) in assay buffer composed by Tris-buffered saline containing BSA and 0.01% thimerosal. Samples were incubated in a 96-well microtiter plate pre-coated with goat anti-rabbit IgG. The assay was based upon the competition between sample aMT6s, and the tracer composed by horseradish peroxidase (HRP)-conjugated aMT6s for a fixed amount of rabbit anti-aMT6s antibody. After 2 h of incubation at 25 °C under agitation (500 rpm), plates were washed four times. Following the addition of the peroxidase substrate 3,3′,5,5′-Tetramethylbenzidine (TMB) and an incubation step of 30 min, the stop solution composed by a strong acid was added and absorbance was measured at l = 450 nm using the MultiSkan FC Spectrophotometer. After correction for the dilution factor, data were reported as ng/mL for subsequent normalization.

#### 4.3.4. ELISA Assay for Urinary Cortisol Determination

Nocturnal and first-morning Cortisol was measured via a direct competitive ELISA kit (Cortisol Express, Cayman Chemical, Ann Arbor, MI, USA), exhibiting suitable accuracy, precision, and sensitivity for our purposes, characterized by cross-reactivity of less than 0.5% with other endogenous steroids and related compounds potentially present in the samples; inter- and intra-assay variations not exceeding, on average, the 10–12% across the expected sample optical density range; and a lower limit of detection (LLOD) of 20 pg/mL. The principle of the assay is based on the competition between free Cortisol and a Cortisol-acetylcholinesterase (AChE, tracer) conjugate for a limited number of monoclonal antibody binding sites by means of a two-step procedure. The day of assay, blanks (non-specific binding and other reagent controls), standards (39.1–5000 pg/mL) and diluted urine samples after thawing were added to the 96-well microplate coated with anti-Cortisol antibodies and incubated for 2 h at room temperature under shaking (600 rpm) (first step). After five washes, a solution containing the AChE substrate plus Ellman’s Reagent was added and incubated in the dark for a further 80–90 m (second step). Ellman’s Reagent or 5,5′-dithio-bis-2-nitrobenzoic acid (DTNB) recognizes and specifically reacts with the thiol group (-SH) of the AChE-deriving product, giving rise to a yellow-colored anion. Absorbance was monitored for 60 min and read within 90 min at 405 nm and 412 nm using the Enspire multimodal reader (PerkinElmer, Waltham, MA, USA). Concentrations were calculated by a semi log 4-PL non-linear regression analysis of the percent of the ratio between Bound in sample and Bound in Blank, % B/B0 values, to obtain Cortisol in pg/mL, then corrected for the dilution factor and subsequently normalized.

### 4.4. Statistical Analysis

All biochemical assays were performed at least in duplicate. Analyte concentrations were interpolated using a 4-PL non-linear regression semi log model [y vs. log(x)] via GraphPad Prism software (Version 10.0, San Diego, CA, USA). Each analyte calibration curve was validated when the resulting R^2^ was >0.995, according to the method validation parameters. The final analyte concentration, calculated as log ng/mL (aMT6s) or log pg/mL (Cortisol) in unknowns, were then transformed into the non-logarithmic measures (x′ = 10^logx^). Cortisol values obtained as pg/mL were then converted into ng/mL. Normalization procedure: To account for interindividual variability in nighttime urine dilution and glomerular filtration rate, the resulting concentrations of aMT6s and Cortisol were then adjusted (C′) using sample specific gravity which normalizes the observed concentration (C_ng/mL_) to a standard specific gravity value (SG) [129]. In the present study we used the SG reference value of 1.020 [130] and the following formula:

C′_ng/mL_ = C_ng/mL_ × [(SG_ref_ − 1.000)/(SG_sample_ − 1.000)]

where C′_ng/mL_ is the normalized urinary concentration, C_ng/mL_ is the measured concentration in each urine sample, SG ref is the chosen reference value [130], and SG-sample is the corresponding specific gravity measured in each urine sample. In this study, SG ref was 1.020 [130], also in accordance with our laboratory averages. C′ normalized concentrations of aMT6s and Cortisol were then multiplied by the total urine volume collected during the approximately 8 h overnight period to obtain total amounts excreted in ng. These values were then converted into the total μg excreted.

A third variable was calculated: the Cortisol/aMT6s ratio (Cort/aMT6s), representing a proxy value of the homeostatic balance between the stress-arousal HPA and rest-relaxing adaptive functions in recruited subjects. We choose to calculate and investigate the Cort/aMT6s ratio instead of the aMT6s/Cort one to rather focus on the Cortisol pathway intrusion during the nocturnal physiological peak of Melatonin. Data were reported as the mean ± SD and mean rank. Chi-square tests were performed to compare categorial socio-demographic variables among groups. Due to the small sample size and the non-normal distribution of data, as obtained by normality and homoscedasticity tests, non-parametric methods were preferred for comparisons and correlations. The Kruskal–Wallis one-way analysis of variance was used to compare socio-demographic variables, psychometric scores, and biochemical markers among the three groups (PTSD, DEP, and CTL), followed by Dunn’s post hoc test with Bonferroni’s correction for multiple testing, as calculated by GraphPad Prism 10.0. For all Kruskal–Wallis comparisons, epsilon-squared (ε^2^) was calculated as a non-parametric measure of effect size.

The correlations between each biochemical parameter with the others and between each biochemical parameter and clinical scores obtained at the psychometric evaluation were carried out using Spearman’s rank correlation coefficient (Rs). Correlations among biological parameters were first carried out in the whole cohort of enrolled subjects following a dimensional approach, then repeated in each group separately, following, in this case, the patients’ stratification. For both biological and clinical correlations, multiple test correction for False Discovery Rate (FDR, Type-I errors) was calculated according to the Graph Pad Prism 10.0 analysis of a stack of *p* values—the Benjamini, Krieger and Yiekutieli method. To appraise the FDR test to our dataset, correlation families were defined based on the biological hypotheses and specific symptom clusters. All correlation analyses were regarded as exploratory and hypothesis-generating, and the Rs Spearman values have been discussed in accordance with Cohen’s criteria [151].

Additionally, statistical significance was set at *p* ≤ 0.05 to curtail Type-II errors potentially occurring when analyzing small-size samples.

## 5. Conclusions

This cross-sectional study suggests the presence in BD patients of alterations at the level of the HPA axis—which reflects allostatic adaptive mechanisms within daily arousal rhythms—and Melatonin production—which represents the main circadian indicator of metabolic rhythms during light–dark transitions—providing original findings regarding the nighttime–early morning period under study. Furthermore, the present results propose that BD patients with PTSD experience significant nighttime distress not only from a clinical point of view but also at the neuroendocrine-molecular level. Under physiological conditions, Cortisol levels are low at night, but under stress and in pathological conditions, an alteration in Cortisol production may occur. The combined evaluation of the neuroendocrine Melatonin pathway, which is dominant during the night, with the low nocturnal activity of HPA axis, suggests herein a neuroendocrine dissociation in BD patients carefully stratified with respect to different clinical features. Hyperarousal and activation of HPA axis in the nighttime was related to the PTSD group, whereas blunted nocturnal Melatonin release was linked to the DEP one, while both correlated with the respective clinical presentation severity. The correlations between Cortisol and aMT6s, along with those involving the CORT/aMT6s ratio, reflected not only the neuroendocrine dysregulation of the two systems, but also a possible alteration in the interplay between them, with different phenotypic profiles in euthymic BD patients with PTSD versus BD patients with MDE without PTSD. The Cort/aMT6s ratio was also found to be associated with patients’ functional impairment, supporting the usefulness in monitoring patients of nighttime urine evaluations. The present results are therefore forerunners of further research to establish the epigenomic [159,160] and metabolomic correlates of these conditions in larger samples of subjects, BD patients and controls. This will contribute to deeply understanding the weight of the observed changes in BD patients at the neuroendocrine/metabolic levels, as well as to identifying biomarkers and therapeutic targets in this clinical population.

## Figures and Tables

**Table 1:** Comparison of urinary parameters in patients with bipolar disorder and Post-Traumatic Stress Disorder (PTSD) or major depressive episode (MDE), and in healthy volunteers (CTL).

Urine Parameters	PTSD, n = 20 Mean ± SD, Mean Rank	DEP, n = 20 Mean ± SD, Mean Rank	CTL, n = 19 Mean ± SD, Mean Rank	H	*p*	ε^2^	Post Hoc
Volume, mL	623 ± 423, 32.15	466 ± 329, 24.32	483 ± 158, 30.44	2.38	0.305	-	-
pH	6.05 ± 0.55, 31.38	6.08 ± 0.56, 32.21	5.72 ± 0.65, 22.97	3.69	0.160	-	-
Specific gravity	1.022 ± 0.009, 29.80	1.022 ± 0.008, 27.37	1.022 ± 0.008, 29.83	0.28	0.870	-	-
Cortisol, μg tot	73.80 ± 70.95, 36.18	24.78 ± 11.89, 20.50	25.97 ± 10.47, 23.18	8.30	0.016	0.113	PTSD > DEP *, PTSD > CTL *
aMT6s, μg tot	19.19 ± 19.5, 31.80	7.80 ± 7.25, 18.89	21.50 ± 17.81, 35.17	10.40	0.006	0.150	DEP < CTL **, DEP < PTSD *
Cortisol/aMT6s	4.53 ± 4.26, 26.41	5.29 ± 3.44, 32.50	2.56 ± 2.59, 18.00	7.44	0.024	0.097	DEP > CTL *

H: Kruskal–Wallis’s test; *p* = statistical threshold; post hoc: Dunn’s multiple comparisons; **, *p* < 0.01; *, *p* < 0.05. Cortisol/aMT6s: The ratio between total overnight urinary Cortisol and aMT6s excretion. Epsilon-squared (ε^2^) values are only reported for statistically significant comparisons (*p* < 0.05).

**Table 2:** Correlations between overnight urine biochemical parameters, HAM-D, YMRS, IES-R and WSAS scores.

Biochemical Parameters	HAM-D	YMRS	IES-R				WSAS				
	Total Score	Total Score	Intrusion	Avoidance	Arousal	Total Score	Work	Home Management	Social Leisure	Private Leisure	Close Relationships
Cortisol, μg	−0.034, *p* = 0.284	−0.028, *p* = 0.294	**0.434,** ***p*** ** = 0.001**	**0.426,** ***p*** ** = 0.004**	**0.429,** ***p*** ** = 0.004**	**0.435,** ***p*** ** = 0.002**	0.089, *p* = 0.186	0.149, *p* = 0.204	0.124, *p* = 0.399	0.068, *p* = 0.663	0.191, *p* = 0.277
aMT6s, μg	**−0.390,** ***p*** ** = 0.002**	**−0.326,** ***p*** ** = 0.008**	−0.058, *p* = 0.237	−0.033, *p* = 0.569	0.038, *p* = 0.547	−0.014, *p* = 0.644	**−0.301,** ***p*** ** = 0.013**	−0.198, *p* = 0.151	−0.198, *p* = 0.225	−0.159, *p* = 0.381	−0.102, *p* = 0.477
Cort/aMT6s ratio	**0.419,** ***p*** ** = 0.002**	**0.339,** ***p*** ** = 0.008**	**0.327,** ***p*** ** = 0.012**	**0.304,** ***p*** ** = 0.036**	0.228, *p* = 0.121	0.277, *p* = 0.291	**0.425,** ***p*** ** = 0.002**	**0.424,** ***p*** ** = 0.004**	0.317, *p* = 0.08	0.279, *p* = 0.154	0.240, *p* = 0.277

Data are presented as Spearman correlation coefficient Rs, *p* = FDR corrected values. **Bold** characters highlight significant correlations, *p* ≤ 0.05. HAM-D, Hamilton Depression Rating Scale; YMRS, Young Mania Rating Scale; IES-R: Impact of Event Scale-Revised; and WSAS: Work and Social Adjustment Scale.

## Data Availability

The data presented in this study are available on request from the corresponding author due to ethical reasons.
